# Flow stability and distribution in horizontal serpentine tube bundles with vertical headers

**DOI:** 10.1016/j.heliyon.2024.e29310

**Published:** 2024-04-09

**Authors:** Viktoria Illyés, Stefan Thanheiser, Felix Ettlinger, Martin Schulz, Markus Haider

**Affiliations:** Institute of Energy Systems and Thermodynamics, TU Wien, Getreidemarkt 9/BA/E302, Vienna, A-1060, Austria

**Keywords:** Thermal energy storage, Fluidization, Particle storage, Density wave oscillation, Maldistribution

## Abstract

Particle-based systems have immense potential for combining thermal energy storage (TES) with renewable energy sources. The so-called sandTES system, which is an active TES system, utilizes sand or other small particles as a storage material and consists of a hot tank, a cold tank, and a reversible fluidized bed heat exchanger. In the preferred design, the tubes are arranged in horizontal serpentine tube bundles; thus, the headers are positioned vertically, for one phase subcritical, two-phase and supercritical water/steam conditions. So far, no design principles have been published for macro-sized vertical headers and horizontal tube bundles. This paper investigates the unbalance in flow distribution and other flow instabilities of such an unusual heat exchanger and its scale-up using APROS simulations.

The steady-state simulations of the tallest investigated heat exchanger show a strong unbalance in the mass flows between individual heat exchanger tubes: the smallest mass flow was only 55% of the largest one when generating steam. Smaller heat exchangers experience less pronounced unbalance factors in the order of 88%. When steam is condensed, the unbalance is insignificant. Dynamic simulations of start-up and shutdown revealed instabilities and that the flow maldistributions even lead to flow reversals in most investigated cases although neither last for stationary operation. Part-load cases show higher unbalances than their respective full-load case by 9 to 17 percentage points. Header design modifications – secondary headers with submodules and orifices – improve the unbalance factor from 55% to 88%.

## Highlights

• A unique heat exchanger with vertical headers is simulated dynamically.
• Flow instabilities occur during transient processes but do not last.
• Vertical headers impact flow distribution significantly.
• Header design modifications improve flow distribution sufficiently.

## Graphical abstract

**Figure:**
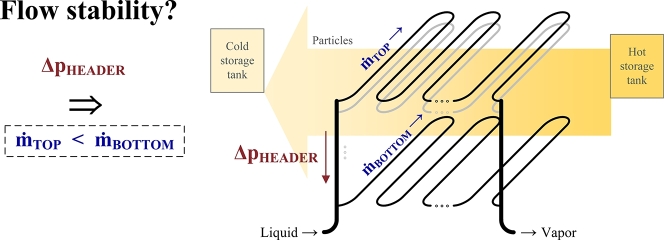

## Nomenclature

***Symbols***
m˙: Mass flow kg/s
$\overset{˙}{Q}$: Heat flow rate W
AR: Header distribution area
F1: Unbalance factor
h: Heat transfer coefficient W/(m^2^ K)
i: Relative nonuniform coefficient
L: Characteristic length m
n: Number of vertical serpentine tubes
p: Pressure bar
S: Absolute nonuniform coefficient
T: Temperature °C
v: Velocity m/s
**G**: Mass flow rate kg/s
**k**: Thermal conductivity W/(m K)
**Nu**: Nusselt number Nu=hL/k
**Pr**: Prandtl number Pr=$c_{p}\mu /k$
**Re**: Reynolds number Re=ρvL/μ
Δp: Pressure difference Pa
μ: Dynamic viscosity Pas
ρ: Density kg/m^3^

**Subscripts**
**approx**: Approximate
**b**: Bulk
**bottom**: Bottom serpentine tube
**ext**: External
**H**: Header
**H2O**: Water
**in**: Inlet
**int**: Internal
**max**: Maximum
**min**: Minimum
**out**: Outlet
**sand**: Sand
**sub**: Subcritical
**super**: Supercritical
**T**: Tubes
**top**: Top serpentine tube
**w**: Wall

**Acronyms**
**HTC**: Heat Transfer Coefficient
**HTF**: Heat Transfer Fluid
**sandTES**: Sand Thermal Energy Storage
**TES**: Thermal Energy Storage

## 1 Introduction

Thermal energy storage (TES) enables renewable energy sources to compete with fossil fuels for base-load capacity. Sensible heat excels at large storage sizes and high temperatures, as sensible-heat materials offer excellent thermal stability. Fluidized bed systems show better heat transfer and thus lead to higher system efficiency than other technologies [2]. Fluidization also makes the operation of active systems possible, as shown in Section 1.1. In active systems, the heat transfer and storage components are separate, so the temperature levels for heat transfer remain constant between the heat storage and transfer media throughout charging and discharging.

The presented TES system, called sandTES, involves a heat exchanger and a storage tank system, with quartz (silicon dioxide, SiO_2_) sand particles as a sensible-heat carrier. The architecture of the heat exchanger specifically requires vertical headers because its particle side is a horizontally moving fluidized bed. The sandTES technology enabling reversible operation with only one heat exchanger is described in Section 1.1.

The present paper summarizes a feasibility study for once-through evaporation and condensation operation (heating and cooling under supercritical conditions) in tube bundles with horizontal serpentines and vertical headers. Through steady-state and dynamic simulations, the paper investigates the flow stability and distribution of the sandTES heat exchanger to judge feasibility of the proposed system, flexibility in part-load operation and potential solutions for arising issues. The focus of the study lies on water as a heat transfer fluid (HTF) in the temperature region with high density changes both in super- and subcritical pressure, i.e., 280-450 °C.

### 1.1 Description of sandTES technology and arrangement of fluidized bed heat exchanger

**Figure 1:**
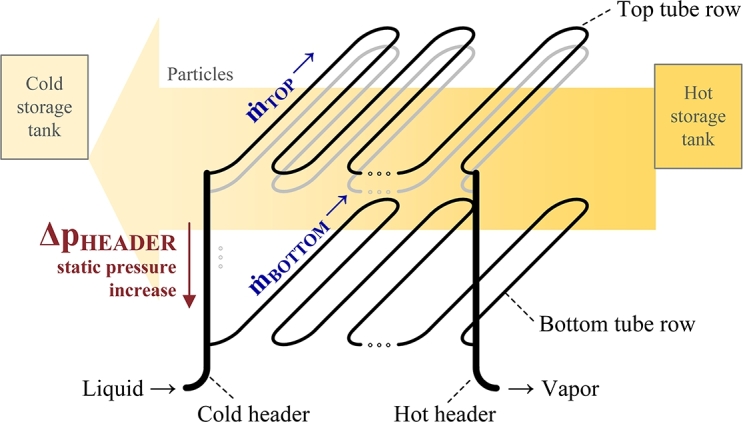
Diagram of fluidized-bed heat exchanger during heating/discharge. The illustration shows the horizontal flow of particles and the orthogonally arranged tubes which enable cross-flow between the particles and the heat transfer medium. The vertical headers result from this arrangement.

The sandTES technology is an active TES system with a particle fluidized-bed heat exchanger and storage. Sand is utilized as a sensible-heat storage material and stored at two temperature levels in a hot tank and a cold tank. During the particle storage charge and discharge, the sand is horizontally moved between the tanks via an air-fluidized bed. The discharge mode, which corresponds to the heating of the heat transfer fluid, is shown in Fig. 1.

The sand flow across the heat exchanger is perpendicular to the tube axes; this design is preferred to other arrangements because it improves heat transfer [36]. Due to the fluidization, the vertical temperature distribution is even, unlike the horizontal temperature distribution from the hot tank to cold tank. Horizontal serpentine rows are stacked vertically, thus requiring vertical headers. A scale-up means adding more rows to the heat exchanger and increasing the total height of the vertical headers.

Compared to the current design, an arrangement with horizontal headers and vertical tube bundles would require the particle flow to move vertically in a moving bed. The heat transfer in fluidized beds is generally higher than in moving beds. Also, a system that uses only one heat exchanger for charge and discharge with moving beds is more complex than our system.

A sandTES heat exchanger can be designed for full flow reversal both on particle side and on water/steam side (charge and discharge of the storage). In such a case, once-through operation both in charge and discharge mode offers the highest simplicity and is therefore the preferred design. In this approach, when the heat exchanger is operating between 280 °C and 450 °C, its hot header contains a low density fluid, while its cold header contains a high density fluid. Under subcritical conditions, the high/low density fluid is water/steam - liquid/gas. Due to the density difference between the hot and cold fluids, static header pressure-drops are more prevalent in the cold header, leading to a nonuniform distribution of pressure drop across the tube rows. The top tube row undergoes the smallest pressure drop along the serpentine tube row, whereas the bottom tube row undergoes the highest one. Hence, the top tube row has a lower mass flow rate than the tube rows below.

### 1.2 Flow instabilities and distribution in heat exchangers

Flow instabilities and maldistributions pose risks to heat exchangers, from performance decline to component failure. In particular, they are listed by Kitto and Stultz, [18]:

• unit control problems, including unacceptable variations in steam drum water level,
• critical heat flux conditions/departure from nucleate boiling/dryout,
• tube metal temperature oscillation and thermal fatigue failure, and
• accelerated corrosion attack.

An uneven flow distribution might lead to insufficient cooling of individual tubes and wall temperatures beyond the safe limits. When only appearing at a lesser degree, the flow maldistribution still influences the efficiency of the heat exchanger. For an efficient supply of steam at similar enthalpies at the end of each tube, a stable and well-defined flow characteristic is necessary for each tube.

Two-phase flow instabilities were first classified by Boure et al. [9]. Instabilities can be static or dynamic. Static instabilities are a series of steady-state conditions where a small perturbation of conditions leads to distinct operating points. These instabilities either lead to a single-event departure to a stable operating point or result in periodic oscillations.

**Figure 2:**
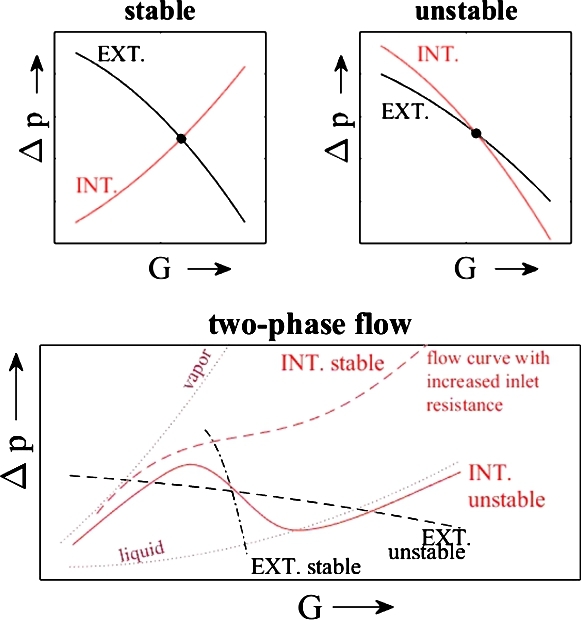
Internal vs. external pressure drop curve of: Top left - stable flow, top right - unstable flow, bottom - two-phase flow with risk of instability has typical “S”-shape.

According to a recent review article on instabilities by Ruspini et al. [30], instabilities related to the characteristic pressure drop curve are among the most widely studied instabilities. These phenomena are also called Ledinegg or excursive instabilities and were published by Ledinegg [19]. A flow is deemed unstable when the internal pressure-drop curve is steeper than the external pressure drop, according to Equation (1):

(1) $$\frac{\partial \Delta p}{\partial G}{|}_{int}\leq \frac{\partial \Delta p}{\partial G}{|}_{ext},$$

where Δ*p* is the pressure loss and *G* is the mass flow rate. The shape of the characteristic curve of a boiling two-phase flow resembles an “S”- (or an “N”-) shape, seen in Fig. 2. The operating conditions on the negative slope of the pressure curve might lead to a Ledinegg instability, depending on the external pressure loss.

A Ledinegg instability may result in flow excursion or even flow reversal. Walter [38] studied flow reversals in natural circulation systems with vertical tubes. The characteristic curves of a tube differ considerably for opposite flow directions. Such opposite flow directions commonly occur during the start-up of these systems. When the system is cold during start-up, the condensing working fluid in its vertical riser tube will stream downwards.

In their recent review paper, O'Neill and Mudawar [27] list Parallel Channel instability as their own distinctive instability relevant to systems with multiple parallel tubes connected via headers: the total flowrate can stay the same while the flow distribution between individual tubes changes drastically. Depending on the causing mechanism, Ledinegg instability or Density Wave Oscillations, this instability is static or dynamic.

**Figure 3:**
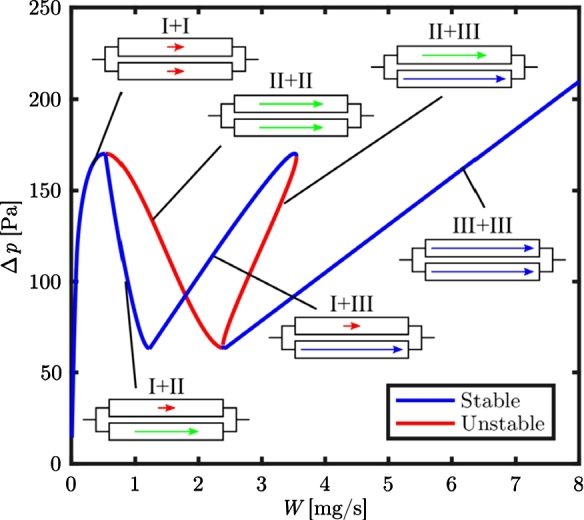
Characteristic pressure drop curve (with total mass flow rate *W*) for two connected parallel channels. Reprinted from International Journal of Heat and Mass Transfer, vol. 107, T. Oevelen, J. Weibel, S. Garimella, Predicting two-phase flow distribution and stability in systems with many parallel heated channels, p. 557-571, 2017, with permission from Elsevier.

The result of Parallel Channel instability is a flow maldistribution shown for two tubes in Fig. 3. When the characteristic pressure drop curve is stable for both individual tubes, then the even distribution is the stable solution for the system, which is true for a very low and a high total mass flow rate. Between these stable solutions, the steady-state flow is maldistributed, meaning one tube sees a higher mass flow than the other. Since it is random in which tubes this happens, there are two solutions. For an increasing number of tubes, there are more solutions. Van Oevelen et al. [37] generalized the eigenvalue problem for an increasing number of parallel channels, in comparison to the analytical and experimental work by other authors, e.g., Baikin et al. [5] and was able to provide maps to determine stable flow conditions for a system with fixed geometry and external pressure curve. A Parallel Channel instability was found to be in the range of 2-100% of the non-uniformity factor or four possible solutions at a macro-sized system with four differently heated tubes at a varied inlet mass flow rate, [42].

Many review papers focus on two-phase flow only. But in supercritical regions, systems close to pseudo-boiling behave similar to two-phase boiling channels, enabling Ledinegg instability and Density Wave Oscillations [4].

Experimental setups and physical models in steady-state are used to understand the mechanisms [5]. For complex systems, researchers use Matlab/Simulink to solve the eigenvalue problem [42], numerical software like RELAP5 (Reactor Leak and Power Safety Excursion), designed for simulation of nuclear reactors [35] or CFD [8].

Changes in flow direction also occur due to negative pressure drops, leading to a single-event departure from the now unstable operating condition to a reversed flow. Therefore, we consider changes in flow direction due to negative pressure drops static instabilities.

The pressure difference between inlet and outlet of the individual tubes varies for all heat exchangers with headers and multiple tubes because of header pressure losses. The result is a nonuniform mass flow distribution, which is sometimes called flow (mal)distribution [25] or unbalance. The pressure drop along headers is a result of the static and dynamic pressure losses of the fluid. For horizontal headers, only the dynamic pressure loss leads to different inlet pressures of the individual tubes, whereas vertical headers also experience a static pressure difference, influencing the tubes' flow characteristics, especially at two-phase or pseudo two-phase flows with high density changes.

Vertical headers have been used previously: Babcock claim in their 1955 book “Steam, Its generation and use” [10] (p. 11-3) that all their straight-tube boilers were made up of sections of staggered tubes, expanded at the ends into vertical headers. But since the first large-scale once-trough boilers in the 1970's, vertical headers disappeared completely and the multitude of parallel evaporator tubes were connected via horizontal headers [16].

The pressure drop in horizontal headers is a well-known aspect during the design of heat exchangers and has been managed since higher gas temperatures at superheaters brought “steam distribution difficulties” in 1922, as Babcock state [10] (p. 12-4). The solution for stabilizing and evening out the flow - now and then - is to include flow resistance devices, see Strauß [34] (p. 196) and Babcock and Wilcox' handbook [10] (p. 12-4).

Compared to horizontal headers, only few studies have been published on vertical headers; the review paper on two-phase flow distribution in parallel channels by Dario et al. [13] from 2013 only cites five other publications (three of them by the same research group). All of them are about microchannels and headers of compact heat exchangers, which are primarily for automotive and air conditioning purposes. In this context, some authors use the term nonuniformities to describe flow maldistribution. Siddiqui et al. [33] published an overview of related studies and their major findings. A generalization of microchannels and macro-sized channels does not represent both systems adequately, as entrance effects and fluid compressibility have to be considered differently [29]. Ong and Thome [28] present transition criteria between micro- and macro-scale systems in the form of a dimensionless Confinement factor.

Flow maldistribution occurs in flat plate solar collectors with single-phase water or water/glycol flow, [31]. Sometimes, headers are inclined. Bava and Furbo [7] investigated the flow distribution in inclined header collectors both numerically and via experiments and found the mass flow rate and viscosity of the fluid to be the main factors influencing the pressure drop and the flow regime in the headers, thereby affecting the flow distribution.

Universally valid criteria for when a flow is too unevenly distributed do not exist in the publically available literature. The interaction of mass flux, pressure drop, and heat input and the resulting consequences are too individual and complex. However, for natural circulation systems, uneven flow distributions have been addressed and researchers have made efforts to give more general guidelines. For example, Walter et al. [38] give maps for the critical heat flux as a function of total heat input and pressure for various cases.

### 1.3 Mitigation options for unstable and maldistributed flows

The operating conditions determine the operating point in the diagram of the characteristic pressure drop curve, 2. By changing the operating conditions, the region of flow instabilities can be avoided. For example, an increased heat input decreases the pressure drop and leads to a more stable flow. An increased overall pressure increases the pressure drop across all heat exchanger tubes, which also makes the flow more stable. The most common, device-level approach for avoiding Ledinegg instabilities is to increase the tube inlet pressure drop, until the form of the characteristic pressure drop curve no longer resembles the “S”-shape and the unstable negative slopes disappear, as shown in Fig. 2 by the dashed internal pressure drop curve. This modification also improves the flow distribution.

The typical flow restriction device for increasing the inlet pressure drop of individual tubes is an orifice, see Kitto and Stultz [18]. Webb and Chung [39] discussed approaches for improved flow distribution and stated that these approaches are highly empirical and effective only for design conditions. They differentiated between flow restrictions at the tube inlets and special distributor devices in the header. Methods for mitigating uneven flow distributions in microchannel heat exchanger headers (manifolds) were also detailed by Siddiqui [33].

In summary, the mitigation options are:

• the proper design of (consecutive) headers with respect to many design and operating parameters (most of which are related to the geometric designs of headers and tubes/channels)
• the use of orifices at the inlet of tubes
• the use of inserts or weirs in the header
• the use of tapered headers (instead of conventional cylindrical ones) with a decreasing diameter at the inlet and an increasing diameter at the outlet header [31]
• the use of bifurcation headers (see Fig. 5 (a) for consecutive and Fig. 5 (b) for bifurcation headers)
• the use of secondary headers with orifices.

Other authors consider about active flow restriction such as valves [26], which is an unfeasible approach for the conditions of big-scale steam generators.

### 1.4 Flow distribution factors

**Figure 4:**
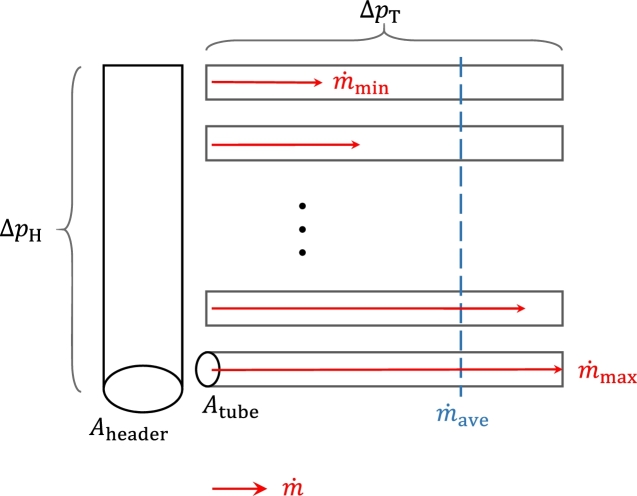
Visualization of inputs to flow distribution factors shown as a header with attached tubes.

Different simple factors have been developed for the design of heat exchangers and the assessment of their flow distribution. These factors are summarized in Table 1, and their inputs are shown in Fig. 4 and discussed below. For the conventional design of superheaters, the common engineering practice is to use a header unbalance factor to quantify the flow maldistribution, which is called unbalance in this context [16]. An unbalance factor defined as:

(2) $$F1=\frac{{\overset{˙}{m}}_{min}}{{\overset{˙}{m}}_{max}},$$

where ${\overset{˙}{m}}_{min}$ and ${\overset{˙}{m}}_{max}$ is the minimum and maximum mass flow rates in one tube, or according to Equation (3)

(3) $$F1_{ave}=\frac{{\overset{˙}{m}}_{min}}{{\overset{˙}{m}}_{ave}},$$

where ${\overset{˙}{m}}_{ave}$ is the average mass flow rate across all tubes, calculated according to Equation (4):

(4) $${\overset{˙}{m}}_{ave}=\frac{1}{n}\sum_{k=1}^{n}{\overset{˙}{m}}_{tube,k}.$$

**Table 1:** Factors for flow distribution.

Unbalance factor	$F1=\frac{{\overset{˙}{m}}_{min}}{{\overset{˙}{m}}_{max}}$
Unbalance factor	$F1_{ave}=\frac{{\overset{˙}{m}}_{min}}{{\overset{˙}{m}}_{ave}}$
Unbalance factor	$F1_{approx}=\sqrt{1-\frac{\Delta p_{H}}{\Delta p_{T}}}$
Flow rate ratio	$R_{i}=\frac{{\overset{˙}{m}}_{tube_{i}}}{{\overset{˙}{m}}_{total}}$
Header-tube distribution area	$A_{R}=\frac{\sum_{n}A_{tube,n}}{A_{header}}$
Absolute nonuniform coefficient	$S^{2}=\frac{1}{n}\sum_{k=1}^{n}{\left({\overset{˙}{m}}_{tube,k}-{\overset{˙}{m}}_{ave}\right)}^{2}$
Relative nonuniform coefficient	$i=\frac{S}{{\overset{˙}{m}}_{ave}}$

**Figure 5:**
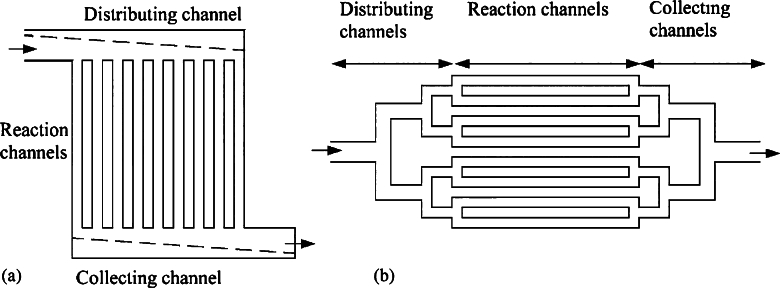
Sketch of geometric arrangement of (a) consecutive headers, (b) bifurcation headers [3].

The following formula is an approximation of Equation (2):

(5) $$F1_{approx}=\sqrt{1-\frac{\Delta p_{H}}{\Delta p_{T}}},$$

where $\Delta p_{H}$ is the maximum header pressure difference and $\Delta p_{T}$ is the pressure drop of the steam in the tubes.

Equation (5) is derived from the solution of the Bernoulli equation between the inlet and outlet of the headers for incompressible fluids and the definition of *F*1 as in Equation (2). The differences in steam densities between the inlet and outlet headers are assumed negligible. Thus, the formula is applicable to superheaters or economizers but unsuitable for designing boilers with two-phase flows. The derivation of Equation (2) is shown in Annex D.

The header pressure difference $\Delta p_{H}$ for single phase flow can be calculated using analytical formulas applying pressure drop characteristics for various heat exchanger arrangements. In the VAIS handbook (former FDBR handbook), one chapter is dedicated to pressure drop and flow dispersion in headers [17] (p. 412-429). While there are analytical formulas to calculate a single phase pressure drop quite easily, two-phase pressure drop is more complex as it is linked to the characteristic flow pattern. For the more complex pressure drop of two-phase systems consisting of more components than plain tubes, numerical methods are usually employed [18].

Some authors use the flow rate to compare the flows in different channels [22] in the form of Equation (6):

(6) $$R_{i}=\frac{{\overset{˙}{m}}_{tube_{i}}}{{\overset{˙}{m}}_{total}},$$

where ${\overset{˙}{m}}_{total}$ is the total mass flow rate among all tubes and ${\overset{˙}{m}}_{tube_{i}}$ is the flow rate of one distinctive tube.

Another, more straightforward design method is the header-tube distribution area $A_{R}$. It is the ratio of the total cross-sectional area of all serpentine tubes to the header cross-sectional area. According to Bajura and Jones [6], the criterion (Equation (7)) for single-phase, horizontal headers for an acceptable flow distribution is:

(7) $$\frac{\sum_{n}A_{tubes,n}}{A_{header}}=A_{R}<1,$$

where $A_{tubes,n}$ and $A_{header}$ are the tube and header cross-sectional area, respectively, and *n* is the number of tubes. García-Guendulain et al. [14] give a more restrictive area ratio of 0.75 for solar collectors.

Two more unbalance parameters should be noted to assess flow distribution: The absolute nonuniform coefficient (or nonuniformity coefficient) is the standard deviation of the mass flow, according to Equation (8),

(8) $$S^{2}=\frac{1}{n}\sum_{k=1}^{n}{\left({\overset{˙}{m}}_{tube,k}-{\overset{˙}{m}}_{ave}\right)}^{2},$$

where ${\overset{˙}{m}}_{tube}$ is the mass flow of the n${}^{th}$ tube row. The relative nonuniform coefficient (Equation (9)) is

(9) $$i=\frac{S}{{\overset{˙}{m}}_{ave}}.$$

In general, the greater the unbalance factor and the smaller the distribution area, absolute/relative and nonuniformity coefficient, the more stable and evenly distributed the flow is.

## 2 Methods

VTT's commercial advanced process simulation software APROS was used in this study because it is a renowned tool for the full-scale industrial modeling of transient operation based on the six-equation model. Using the six-equation model, APROS solves the mass, momentum, and energy balances for the gaseous and fluid phases separately and, therefore, shows an excellent representation of the two-phase flow behavior [1]. APROS can detect static instabilities and even dynamic flow instabilities, such as density wave oscillations [20].

Since APROS uses heat transfer and pressure drop correlations, the results are only as good as the underlying models. Due to the high number of validations of the six-equation model under many thermal hydraulic separate effect tests (steady-state cases) and integral tests (dynamic cases) at similar operating conditions as needed in this study [40], the software is expected to deliver reliable results.

### 2.1 Water/steam side

The Mokry correlation [23], Equation (10), was used in APROS to calculate the heat transfer in supercritical operation:

(10) $$Nu_{super}=0.0061Re^{0.904}Pr^{0.658}{\left(\frac{\rho_{w}}{\rho_{b}}\right)}^{0.564},$$

where $\rho_{w}$ and $\rho_{b}$ are the water densities at the wall and the bulk.

For the subcritical heat transfer, the “Dittus-Boelter correlation” was used, with these coefficients originally published by McAdams [21], according to Equation (11):

(11) $$Nu_{sub}=0.023Re^{0.8}Pr^{0.4}.$$

### 2.2 Particle/fluidized bed side

**Figure 6:**
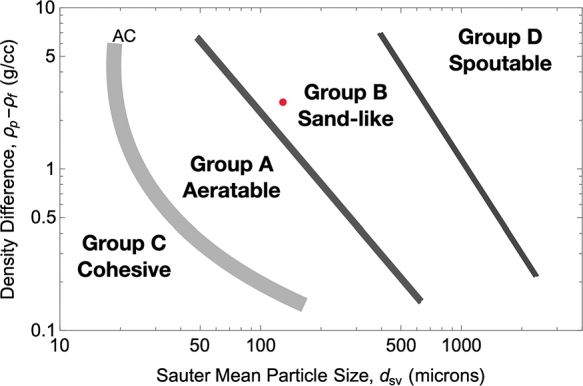
“Interpretation of Geldart groups”, adapted from a figure by Cocco and Chew [12] under the CC BY license. Red dot indicates particles used for this study (150 μm and 2650 kg/m^3^).

The fluidized bed was modeled in APROS as a custom fluid on the heat exchanger's shell side. The specific heat capacity of the quartz (SiO_2_) sand particles was modeled as a function of temperature based on the properties given in NIST [11]. The chosen sand particles of a uniform particle size of 150 μm fall into Geldart [15] group B, close to AB, see Fig. 6.

The investigated tube bundle included helical fins on the tubes' outer surface. To accurately predict the heat transfer between the fluidized bed and the tube surface, the experimental findings by Thanheiser [36] were used, performed for several mean particle sizes and tube bundle / sand flow configurations at about 40 °C bed temperature. These findings yielded a heat transfer coefficient (HTC) of approximately 1200 W/(m^2^ K) between the fluidized bed and the plain tube surface (i.e., not accounting for the fin's contribution to the heat transfer surface – the so-called virtual HTC). Zabrodsky's [41] correlation for predicting the maximum achievable HTC in a fluidized bed was then used to scale the result from the original 40 °C bed temperature to the operating temperature of the fluidized bed during the simulations. Although Zabrodsky's correlation is dimensionally inconsistent and only indirectly includes the particles' specific heat capacity through its dependence on particle density, it correctly represents the dependence of the HTC on gas thermal conductivity with the power 0.6 [24] (p. 90-91). Equation (12) should therefore hold in scaling the HTC to higher temperatures.

(12) HTC=1200W/(m2K)(kairkair,40C∘)0.6,

where $k_{air}$ is the thermal conductivity of the fluidization gas (dry air) at the simulated bed temperature and $k_{air,40}$ is the thermal conductivity of dry air at 40 °C.

## 3 Case study

This case study combines conditions grouped into four categories:

• **Heat exchanger design**: Basic, Basic Advanced, Triple, Triple Advanced
• **Operation mode**: Heating (storage discharge), Cooling (storage charge)
• **Load**: Full-load, Part-load, Start-up, Shutdown
• **Pressure**: Supercritical, Subcritical

Most of the simulated cases feature the basic heat exchanger design *Basic*. The *Triple* heat exchanger is thrice as tall as *Basic* and thus undergoes a larger static pressure increase over the header height. Both designs have advanced versions (*BasicA* and *TripleA*), where header design modifications shift the determining pressure loss to the tubes. The heat exchanger designs are described in Section 3.2.

The heat exchanger is operated in two modes to enable charging and discharging of the particle storage. During heating (storage discharge), cold water enters the heat exchanger and cools down the hot particles. Vice versa, during cooling (storage charge), hot water enters the heat exchanger and heats to cold particles.

Stationary working conditions for the heat exchanger are full-load and part-load. Simulations are conducted until the steady state is reached. Dynamic simulations show the start-up and shutdown processes of the heating operation, in which the sand and water inlet temperatures are kept constant while the mass flow rate increases and decreases from 0% to 100% start-up and from 100% to 0% shutdown of the nominal load over 1 h. The initial temperature for start-up is 25 °C. The full operation steady-state case gives the initial conditions for the shutdown process.

Section 3.1 explains the cases' combinations of conditions. The exact simulated conditions in each case are in Annex C Simulated cases.

### 3.1 Simulated cases

**Figure 7:**
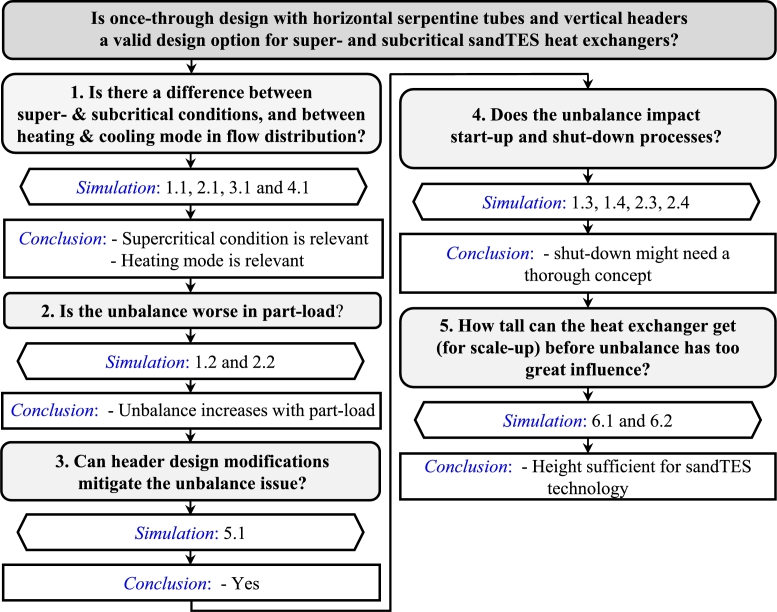
Flow of decision-making on cases to simulate.

Out of the 64 possible combinations of heat exchanger design, operation mode, load and pressure (super- or subcritical), 13 cases are chosen to narrow down the area of importance. The decision-making process is illustrated in Fig. 7. The first three rounds of simulations are performed on the *Basic* design. A test of all combinations of operation modes and pressures under full-load shows that the supercritical pressure and the heating operation are more relevant than the other options. The part-load condition is examined at both sub- and supercritical pressures, in heating mode and for *Basic*. As for the advanced designs, it is determined whether header design modifications can mitigate the unbalance.

The simulated cases are summarized in Table 2.

**Table 2:** Simulated cases and their specifications. For abbreviations and details, see Table 3 in Annex A.

Case	Superc	Subc	Heat	Cool	FL	PL	UP	DOWN	Basic	BasicA	Triple	TripleA
1.1	x		x		x				x			
1.2	x		x			x			x			
1.3	x		x				x		x			
1.4	x		x					x	x			

2.1		x	x		x				x			
2.2		x	x			x			x			
2.3		x	x				x		x			
2.4		x	x					x	x			

3.1	x			x	x				x			

4.1		x		x	x				x			

5.1	x		x		x					x		

6.1	x		x		x						x	
6.2	x		x		x							x

### 3.2 Heat exchangers and header design modifications

A tube bank with a staggered tube arrangement and helical fins is used, in either a regular or an advanced variant. The regular design encompasses horizontal serpentine tubes, stacked vertically and connected by vertical headers (as explained above.)

**Figure 8:**
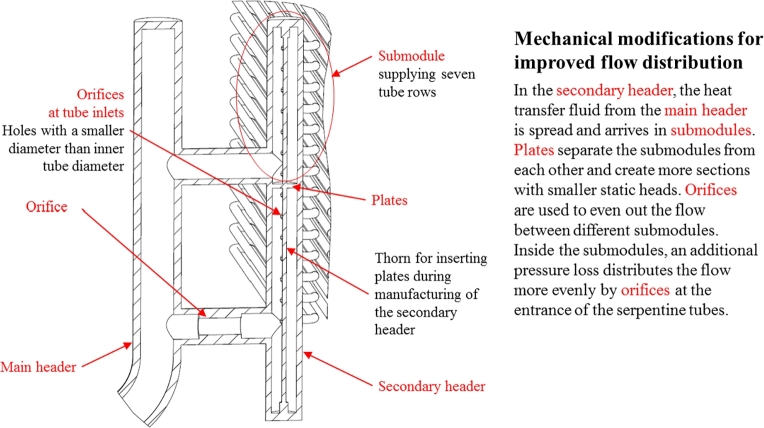
Header design modifications for improved flow distribution of *BasicA*.

In the advanced design, the regular vertical headers are replaced by special ones, shown in Fig. 8. Since the headers are large diameter pressure vessels with high wall thickness, the adaptations are kept be as minimal and easy to fabricate as possible. The design includes flow restrictions to even out the flow and plates to limit the effective static pressure head on individual tubes, making a secondary header necessary.

Two advanced designs are investigated: *BasicA* and *TripleA* (thrice as tall as *Basic*), which feature the following header design modifications for improved flow distribution:

• a secondary header made up of submodules separated by plates,
• orifices at the serpentine tube inlets,
• orifices in the connecting tubes between main and secondary header. This feature is only included in the heat exchanger's cold header.

The mechanical concept of *BasicA* is shown in Fig. 8. The geometrical parameters are listed in the attachment in Annex B Heat exchanger geometrical data.

## 4 Results and discussion

### 4.1 Steady-state temperature distribution

**Figure 9:**
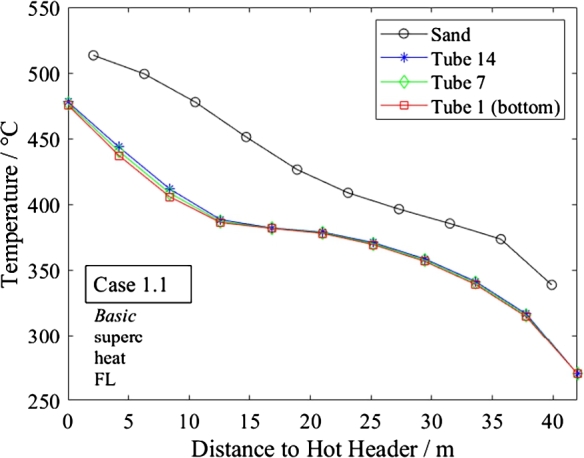
Steady-state temperature distribution of sand and water in different tubes in supercritical heating mode under full-load (Case 1.1, *Basic*).

**Figure 10:**
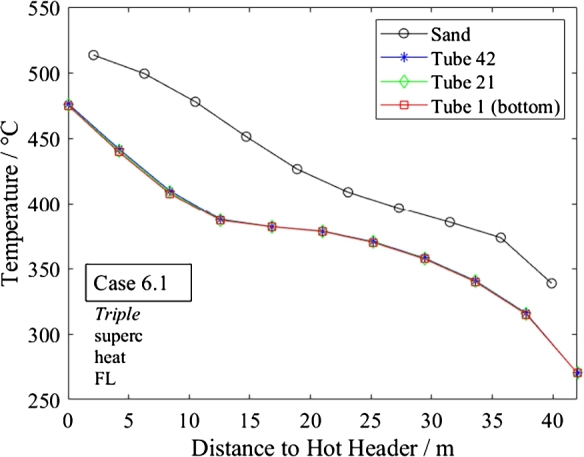
Steady-state temperature distribution of sand and water in different tubes in supercritical heating mode under full-load.

Figure 9, Figure 10 show the temperature distributions for the sand and water in the bottom, middle and top tube in *Basic* and *Triple* in supercritical heating mode. Both designs meet the total heat transfer requirements well. Furthermore, in the steady-state, no significant difference in temperature is seen between the bottom and top tubes although the flow is distributed imperfectly. The advanced design does not appear to provide a significant improvement compared to the regular design in steady state conditions.

### 4.2 Steady-state flow distribution

**Figure 11:**
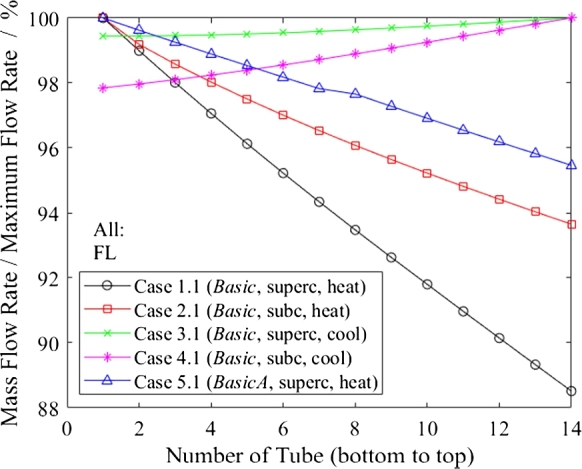
Mass flow rate in relation to maximum flow rate as function of tube number in full-load cases.

In Fig. 11, the flow distributions of several full-load cases are shown with the unbalance factor according to Equation (2). During heating (Cases 1.1, 2.1 and 3.1), the bottom tube has the highest mass flow rate, which was expected since cold, high-density water is supplied to the inlet header from the bottom.

The supercritical heating mode of Case 1.1 shows the worst flow distribution, whereas the subcritical heating mode (Case 2.1) has a much more even distribution. One might think that the density difference between hot and cold headers is higher at subcritical pressures than at supercritical pressures, leading to a larger pressure drop in the cold header. Nevertheless, the supercritical operation is more challenging and the reason lies in the greater proportion of header pressure losses in relation to total pressure loss across the heat exchanger (16.9% in supercritical vs. 7.6% in subcritical operation). The determining pressure loss in subcritical operation occurs in the two-phase flow in the tube bundle (28.1 kPa in supercritical vs. 60.5 kPa in subcritical operation), and not in the headers (5.7 kPa in supercritical vs. 5.0 kPa in subcritical operation). Therefore, the effect of the header pressure loss does not have the same impact as in supercritical operation. The pressure loss across the header does influence the individual tubes to a higher degree when a greater share of the total pressure drop comes from the header in comparison to the tube bundle. The importance of the pressure losses ratio comes as no surprise, since it is also found in the equation for the approximate unbalance factor, see Equation (5).

All cooling simulations show a higher mass flow rate in the top tube. Contrary to the heating mode, where the static pressure loss adds to the pressure loss of the (inlet) header, the static pressure loss reduces the outlet pressure loss of the cold header. Hence, the lower tubes have a higher back pressure due to the static pressure at the outlet. As a result, the flow distribution is better in cooling mode, as shown by comparisons of Case 1.1 with 3.1 and Cases 2.1 and 4.1 in Fig. 11. Cases 3.1 and 4.1 should not be used to compare supercritical and subcritical cooling directly, as the water mass flow in the subcritical case (4.1) is almost half that in the supercritical case (3.1). Implementing more flow restrictions in the cold header to even out the heating mode distribution will negatively impact the cooling mode.

Another factor in the difference between heating and cooling is that the inlet header's inlet is placed at its bottom. Hence, in heating mode, the cold header's inlet is closer to the tubes prone to experiencing the higher mass flow rate (those at the bottom), whereas during cooling, the hot header's inlet is farther away from the upper tubes with more mass flow rate. CFD showed the streamlines in headers and explain this phenomenon [32]. Placing the header's in- and outlets at their tops is an option to improve the flow distribution for the heating mode, but this would negatively affect cooling.

**Figure 12:**
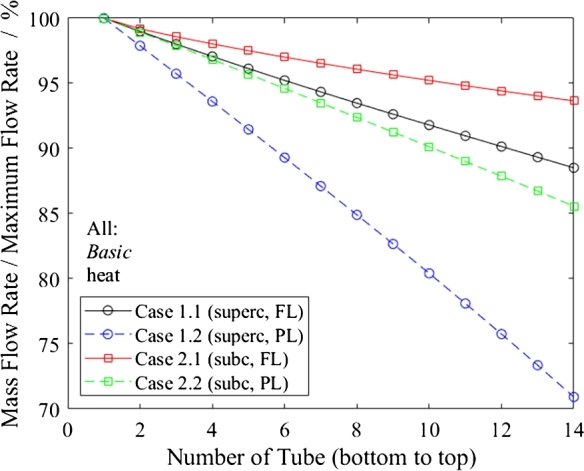
Mass flow rate in relation to maximum flow rate as function of tube number for comparison of part-load (dashed lines) and full-load cases.

Part-load operation generally results in increased flow maldistribution; Fig. 12 shows a comparison of the full-load Cases 1.1 and 2.1 with the part-load Cases 1.2 and 2.2. The reason is that the tube bundle pressure loss significantly reduces while the header pressure losses stay the same. This fact penalizes either the flexibility of the system, as part-load is limited, or the efficiency in full load if the flow restrictions were designed to accommodate the part-load case as the design criterion and cause a higher pressure loss than necessary in full load.

**Figure 13:**
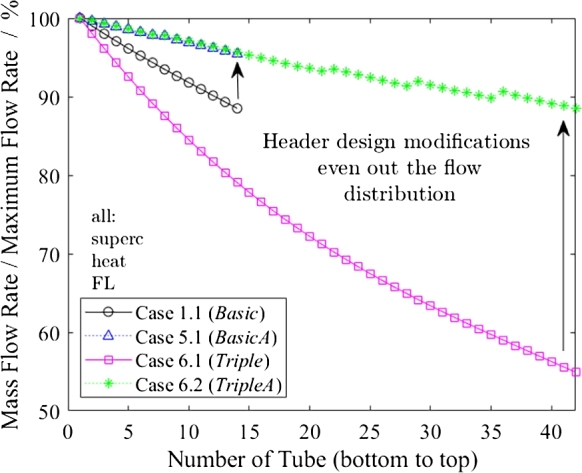
Mass flow rate in relation to maximum flow rate as a function of tube number for comparison of heat exchanger designs.

Fig. 13 depicts the influence of the overall height of the heat exchanger on flow distribution through a comparison of *Basic* (Case 1.1) with *Triple* (Case 6.1). The *Basic* flow distribution is almost linear, whereas the *Triple* presents a quadratic trend. The linear trend can be explained by the linear relationship for the static head while dynamic pressure losses are negligible. The dynamic pressure losses in long headers, such as those in the *Triple* case, cannot be neglected and lead to the linear dependency being overlapped by a quadratic one.

### 4.3 Influence of header design modifications on flow distribution

The studied header design modifications even out the flow distribution substantially, as indicated by the enhancement from Case 1.1 (*Basic*, no improvements) to 5.1 (*BasicA*, with improvements) and from Case 6.1 (*Triple*) to 6.2 (*TripleA*) in Fig. 13. For Case 6.2, the presence of the submodule results in jumps in the mass flow rate between the bottom tube of one submodule and the top tube of the submodule below. Within the submodules, the flow distribution trend mainly shows a linear dependency due to the static pressure loss.

Scaling-up to such a tall height as that of *Triple* requires these modifications or similar measures in order to ensure proper flow distribution.

The downside of these header design modifications is the additional pumping power needed to compensate for the increased pressure losses. The pressure loss across the whole heat exchanger doubles for *BasicA* (Case 5.1) compared with *Basic* (Case 1.1). For the *Triple* configuration, the pressure loss of the heat exchanger with header design modifications (*TripleA*, Case 6.2) is higher by a factor of nearly 1.5 compared with that of the heat exchanger without header design modifications (*Triple*, Case 6.1).

### 4.4 Parameters for evaluation of flow distribution

**Figure 14:**
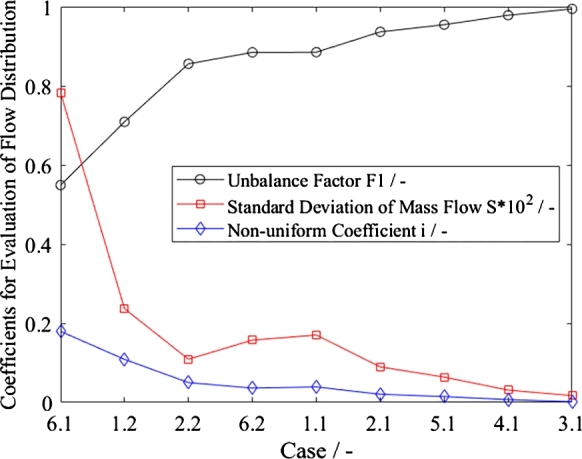
Parameters for evaluation of flow distribution, see Table 2 for case descriptions.

Three important parameters for evaluating flow distribution are presented in Figs. 14, and they show comparable trends. Only the standard deviations indicate that the mass flows of Case 1.1 (*Basic*) and Case 6.2 (*TripleA*) are less evenly distributed than those of the part-load Cases 1.2 and 2.2. Since the calculation of all these parameters is based on the same inputs, it can be concluded that the unbalance factor *F*1 may also be used for the sake of simplicity, even in two-phase or pseudo-two-phase (supercritical) configurations.

The area ratio $A_{R}$ is 0.35 for the *Basic* and 1.04 for the *Triple* heat exchanger. Although the value for the *Basic* heat exchanger is even lower than the value of 0.75 recommended by García-Guendulain [14], the flow maldistribution is significant, for example in Case 1.1. Also, the area ratio cannot represent the changed conditions of different cases and the advanced design. Therefore, the area ratio is not recommended as a design criterion for the sandTES heat exchanger.

### 4.5 Start-up and shutdown processes

**Figure 15:**
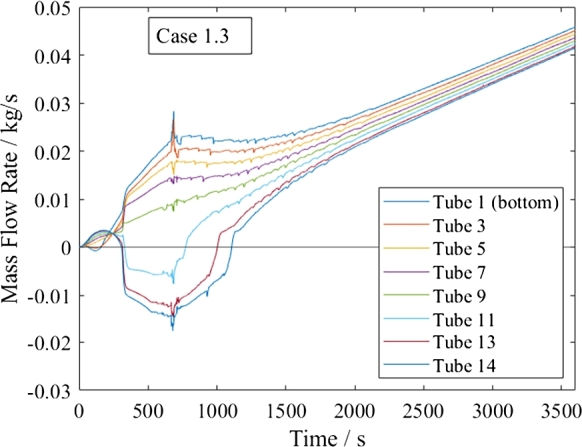
Start-up process in supercritical heating mode for *Basic*.

**Figure 16:**
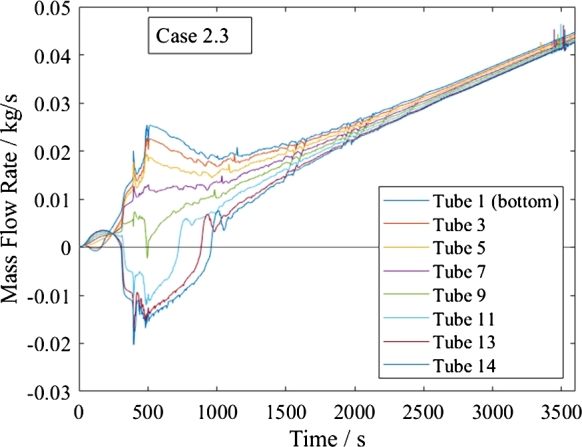
Start-up process in subcritical heating mode for *Basic*.

All start-up and shutdown processes involve a change in flow direction in the upper tubes, which results in recirculation in the heat exchanger, as shown in Figure 15, Figure 16 exemplarily. This change is due to the reduction in the upper tubes' pressure drop caused by the increasing ratio between the cold and hot header pressure drop, which even reaches negative values. This flow excursion fits into the group of static Parallel Channel instability, although it is not caused by a Ledinegg instability nor by Density Wave Oscillations. The maldistribution with reversing flow is more severe than what was found in the literature for Parallel Flow instability. Such an instability was found to be in the range of 2-100% of the non-uniformity factor for four possible solutions at a macro-sized system with our differently heated pipes at a varied inlet mass flow rate, [42].

In all cases, the negative mass flow does not last longer than 20 min into the start-up process and the heat exchanger's operation stabilizes.

**Figure 17:**
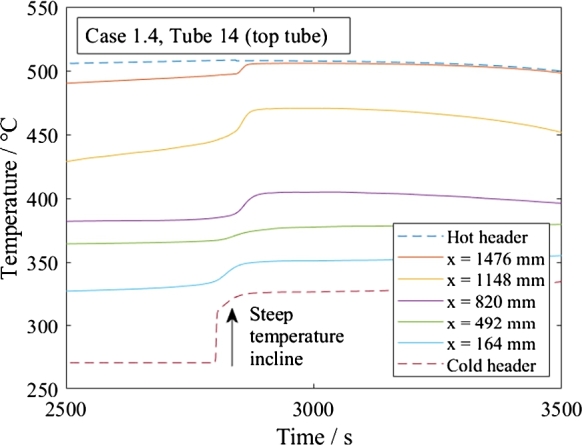
Temperatures in top tube during shutdown supercritical cooling mode. The temperatures correspond to x-coordinates (horizontal distance to the cold header).

The recirculation might become problematic if fluid from the hot header flows back as is happening during shutdown. Drastic temperature changes occur as hot fluid travels back the tube and enters the cold header without being cooled enough. This case is simulated for *Basic* under shutdown of the subcritical cooling mode, as seen in Fig. 17. The temperature in the cold header rises steeply; the temperature increase rate is 41.7 K/s, and the maximum temperature is approximately 326 °C, which is 56 K above the stationary conditions of 273 °C. Especially for pressure vessels with a high wall thickness, quick temperature changes are usually not allowed to prevent thermal stresses and failure of the component. For industrial realization, this topic will need further detailed analysis. At least, the design temperature of the tube and the inlet header need to be specified to the hot outlet condition, and the header/tube design needs to be analyzed for thermal stresses. Alternatively, the shutdown process should be designed carefully. Currently, the water flow rate and sand flow rates are reduced to become zero after 3600 s.

**Figure 18:**
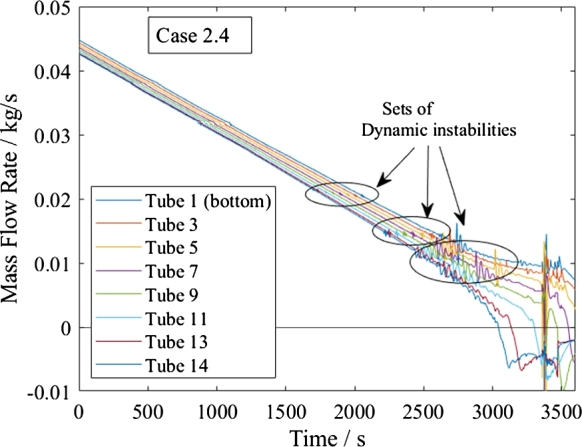
Mass flow rates during shutdown of subcritical heating mode.

The subcritical cases undergo dynamic instabilities, as depicted by Figure 16, Figure 18. These Density Wave Oscillations occur under similar conditions in every tube. Their period is well within the range of 1.5-2 reported in literature, [27]. When appearing in one tube, such an instability only slightly influences the other tubes and does not lead to a Parallel Channel instability. The instabilities are more pronounced during shutdown than start-up but do not last in either.

**Figure 19:**
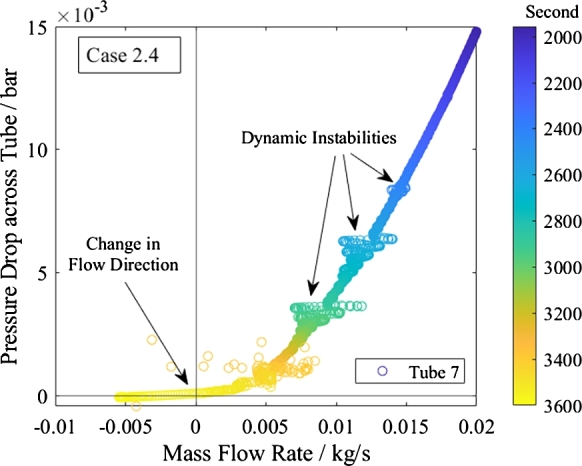
Characteristic pressure curve of top tube of shutdown of subcritical heating mode.

**Figure 20:**
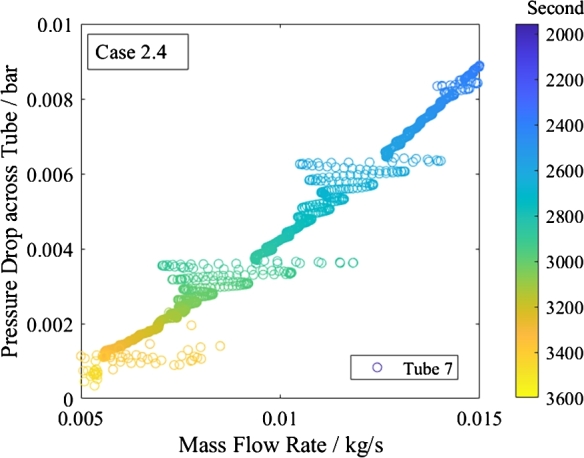
Detail of mass flow rates during shutdown of subcritical heating mode.

Figure 19, Figure 20 present the characteristic pressure drop curve of the middle tube. The mass flow rate oscillates at the imposed pressure drop. The trend at zero pressure drop and mass flow rate is smooth and does not indicate any previously defined instability.

## 5 Conclusion

The present paper summarizes a feasibility study for once-through operation of horizontal serpentine tube bundles with vertical headers. Horizontal serpentine tube bundles are considered a more flexible and efficient arrangement for fluidized bed heat exchangers than vertical serpentines of horizontal tubes, which are feasible but require more piping effort and are less efficient when using moving beds instead of fluidized beds.

Steady-state simulations via APROS show that flow distribution is more challenging in supercritical operation because the determining pressure loss does not occur in the tube bundle the way it does in the subcritical case. The heating mode (steam generation), which corresponds to particle storage discharge, has a more uneven flow distribution than the cooling mode. The unbalance factor is a reasonable parameter for assessing the distribution, even for conditions with strong density change. As expected, the unbalance factor has the lowest value of around 80% for the tallest heat exchanger, highlighting the effect of the static pressure losses in the headers.

The sandTES heat exchanger design with horizontal serpentines of horizontal tubes combined with vertical headers can be upscaled by integrating header design modifications to improve its flow distribution. Although the part-load flexibility is penalized by the vertical headers, the system still may be worth it when part-load is more the exception than the use case. Conventional once-through systems sometimes have recirculation systems to achieve low part-load requirements which could also be an option for the current system.

Dynamic instabilities occur during simulation of the start-up/shutdown transient processes, which confirms that APROS is an appropriate tool for problems of this kind. Close attention to the design of shutdown processes is required as recirculation is likely to happen. The recirculating of hot fluid leads to a temperature increase along the tube and might even reach the cold header.

As a summary, the study has shown that once-through design with vertical headers is feasible even for large heat exchangers, but definitely requires special header design that results in a slightly higher pressure drop of the whole heat exchanger and therefore requires an increased pump capacity. The authors recommend numerical software like APROS for the design process.

Due to the niche application of macro-sized vertical headers as of today, a general approach of addressing the distribution issue in such heat exchangers, similar to what has been done for natural circulation problems, is desirable but not likely. The next step for a specific heat exchanger is optimizing the control strategy for the dynamic processes. Although APROS is an exceptional tool, experimental data would further advance the understanding of an unbalance in horizontal serpentine tube bundles with vertical headers.

## CRediT authorship contribution statement

**Viktoria Illyés:** Writing – review & editing, Writing – original draft, Formal analysis. **Stefan Thanheiser:** Writing – review & editing, Supervision. **Felix Ettlinger:** Software. **Martin Schulz:** Software. **Markus Haider:** Writing – review & editing, Supervision, Funding acquisition.

## Declaration of Competing Interest

The authors declare the following financial interests/personal relationships which may be considered as potential competing interests: Markus Haider has patent ##WO2012027769A2 licensed to 10.13039/501100004729TU Wien. Markus Haider has patent ##WO2015172172A4 licensed to 10.13039/501100004729TU Wien. Markus Haider has patent ##WO2015188214A1 licensed to 10.13039/501100004729TU Wien. Markus Haider has patent ##WO2017210713A1 licensed to 10.13039/501100004729TU Wien. Markus Haider has patent ##WO2020097657A1 licensed to 10.13039/501100004729TU Wien. sandTES technology is licensed to Andritz AG, Austria.

## Figures and Tables

**Table 3:** Abbreviations used in Table 2.

Abb.	Title	Short description
Superc	Supercritical	Supercritical pressure at water side (single-phase heat transfer and pressure loss), p= 242 bar
Subc	Subcritical	Subcritical pressure at water side (two-phase heat transfer and pressure loss), p= 141 bar

Heat	Heating	Heating mode of the heat exchanger, corresponds to discharge of the sand storage
Cool	Cooling	Cooling mode of the heat exchanger, corresponds to discharge of the sand storage

FL	Full-load	Steady state conditions in full-load
PL	Part-load	Steady state conditions in part-load, 50% of full-load mass flow rate
UP	Start-up	Dynamic process of start-up
DOWN	Shut-down	Dynamic process of shut-down

Basic	Basic	*Basic* heat exchanger design, height=0.7 m, number of horizontal tube rows = 14
BasicA	Basic Advanced	*Basic* heat exchanger design with submodules and orifices to homogenize mass flow distribution
Triple	Triple	Heat exchanger design *Triple*, triple the height of *Basic*, height= 2.1 m, number of tube rows = 42
TripleA	Triple Advanced	Heat exchanger design *Triple* with submodules and orifices to homogenize mass flow distribution

**Table 4:** Heat exchanger designs.

Basic, BasicA	
Bundle height / m	0.7
Number of vertical tube rows / -	14

Triple, TripleA	

Bundle height / m	2.1
Number of vertical tube rows / -	42

**Table 5:** Heat exchanger parameters.

Header parameters	
Outside diameter / mm	114
Wall thickness / mm	18

Bundle parameters	

Bundle length / mm	1640
Bundle depth / mm	960
Vertical pitch / mm	50
Horizontal pitch / mm	40

Tube parameters	

Outside diameter / mm	20
Wall thickness / mm	3.6
Number of bends / -	41
Serpentine tube length / mm	42000

Fin parameters	

Height / mm	10
Thickness / mm	2
Pitch / mm	9

Submodules	

Secondary header outer diameter / mm	86
Secondary header wall thickness / mm	18
Orifice diameter / mm	40
Orifice length / mm	100
Orifice diameter at tube inlets / mm	5

**Table 6:** Case 1.1.

Mode	-	Heating
Load	-	Full-load
H_2_O conditions		

Supercritical pressure at inlet	$p_{H2O}^{in}$ / bar	241.8
Inlet temperature	$T_{H2O}^{in}$ / °C	270.3
Outlet temperature	$T_{H2O}^{out}$ / °C	475.5
Mass flow rate	${\overset{˙}{m}}_{H2O}$ / kg/s	0.61
Pressure difference over heat exchanger	Δ*p*_*H*2*O*_ / Pa	28123.9

Sand conditions		

Inlet temperature	$T_{sand}^{in}$ / °C	513.4
Outlet temperature	$T_{sand}^{out}$ / °C	338.6
Mass flow rate	${\overset{˙}{m}}_{sand}$ / kg/s	5.77

Geometry		

Heat exchanger design	-	Basic
Vertical rows	*n* / -	14
Horizontal rows	*m* / -	42
Modifications	-	No

Results		

Transferred heat	$\overset{˙}{Q}$ / kW	1158.3
Unbalance factor	*F*1 / -	0.8851
Absolute nonuniform coefficient	*S* / -	0.0017
Relative nonuniform coefficient	*i* / -	0.0391

**Table 7:** Case 1.2.

Mode		Heating
Load		Part-load
H_2_O conditions		

Supercritical pressure	$p_{H2O}^{in}$ / bar	241.6
Inlet temperature	$T_{H2O}^{in}$ / °C	270.3
Outlet temperature	$T_{H2O}^{out}$ / °C	497.8
Mass flow rate	${\overset{˙}{m}}_{H2O}$ / kg/s	0.61
Pressure difference heat exchanger	Δ*p*_*H*2*O*_ / Pa	10015.5

Sand conditions		

Inlet temperature	$T_{sand}^{in}$ / °C	513.4
Outlet temperature	$T_{sand}^{out}$ / °C	330.1
Mass flow rate	${\overset{˙}{m}}_{sand}$ / kg/s	2.885

Geometry		

Heat exchanger design	-	Basic
Vertical rows	*n* / -	14
Horizontal rows	*m* / -	42
Modifications	-	No

Results		

Transferred heat	$\overset{˙}{Q}$ / kW	605.8
Unbalance factor	*F*1 / -	0.7091
Absolute nonuniform coefficient	*S* / -	0.0024
Relative nonuniform coefficient	*i* / -	0.1087

**Table 8:** Case 1.3.

Mode	-	Heating
Load	-	Start-Up
Duration	*t* / h	1
End conditions	-	Case 1.1
H_2_O start conditions		

Supercritical pressure	$p_{H2O}^{in}$ / bar	241.5
Temperature	*T*_*H*2*O*_ / °C	25.0
Mass flow rate	${\overset{˙}{m}}_{H2O}$ / kg/s	0.000

Sand start conditions		

Temperature	*T*_*sand*_ / °C	25.0
Mass flow rate	${\overset{˙}{m}}_{sand}$ / kg/s	0.000

Geometry		

Heat exchanger design	-	Basic
Vertical rows	*n* / -	14
Horizontal rows	*m* / -	42
Modifications	-	No

Results		

Ledinegg Instabilities	-	No
Flow Reversals	-	Yes

**Table 9:** Case 1.4.

Mode	-	Heating	
Load	-	Shut-down	
Duration	*t* / h	1	
Start conditions	-	Case 1.1	
H_2_O end conditions			

Supercritical pressure	$p_{H2O}^{in}$ / bar	241.5	
Inlet temperature	$T_{H2O}^{in}$ / °C	270.3	
Outlet temperature	$T_{H2O}^{out}$ / °C	491.7	
Mass flow rate	${\overset{˙}{m}}_{H2O}$ / kg/s	0.000	

Sand end conditions			

Inlet temperature	$T_{sand}^{in}$ / °C	513.4	
Outlet temperature	$T_{sand}^{out}$ / °C	337.9	
Mass flow rate	${\overset{˙}{m}}_{sand}$ / kg/s	0.013	

Geometry			

Heat exchanger design	-	Basic	
Vertical rows	*n* / -	14	
Horizontal rows	*m* / -	42	
Modifications	-	No	

Results			

Ledinegg Instabilities	-	No	
Flow Reversals	-	Yes	

**Table 10:** Case 2.1.

Mode		Heating
Load		Full-load
H_2_O conditions		

Subcritical pressure	$p_{H2O}^{in}$ / bar	140.6
Inlet temperature	$T_{H2O}^{in}$ / °C	270.3
Outlet temperature	$T_{H2O}^{out}$ / °C	489.9
Mass flow rate	${\overset{˙}{m}}_{H2O}$ / kg/s	0.61
Pressure difference heat exchanger	Δ*p*_*H*2*O*_ / Pa	60585.0

Sand conditions		

Inlet temperature	$T_{sand}^{in}$ / °C	513.4
Outlet temperature	$T_{sand}^{out}$ / °C	320.0
Mass flow rate	${\overset{˙}{m}}_{sand}$ / kg/s	5.77

Geometry		

Heat exchanger design	-	Basic
Vertical rows	*n* / -	14
Horizontal rows	*m* / -	42
Modifications	-	No

Results		

Transferred heat	$\overset{˙}{Q}$ / kW	12734.8
Unbalance factor	*F*1 / -	0.9365
Absolute nonuniform coefficient	*S* / -	0.00090
Relative nonuniform coefficient	*i* / -	0.0206

**Table 11:** Case 2.2.

Mode	-	Heating
Load	-	Full-load
H_2_O conditions		

Subcritical pressure	$p_{H2O}^{in}$ / bar	140.6
Inlet temperature	$T_{H2O}^{in}$ / °C	270.3
Outlet temperature	$T_{H2O}^{out}$ / °C	508.4
Mass flow rate	${\overset{˙}{m}}_{H2O}$ / kg/s	0.305
Pressure difference heat exchanger	Δ*p*_*H*2*O*_ / Pa	20328.5

Sand conditions		

Inlet temperature	$T_{sand}^{in}$ / °C	513.4
Outlet temperature	$T_{sand}^{out}$ / °C	312.7
Mass flow rate	${\overset{˙}{m}}_{sand}$ / kg/s	2.885

Geometry		

Heat exchanger design	-	Basic
Vertical rows	*n* / -	14
Horizontal rows	*m* / -	42
Modifications	-	No

Results		

Transferred heat	$\overset{˙}{Q}$ / kW	659.2
Unbalance factor	*F*1 / -	0.8557
Absolute nonuniform coefficient	*S* / -	0.0011
Relative nonuniform coefficient	*i* / -	0.0501

**Table 12:** Case 2.3.

Mode	-	Heating
Load	-	Start-Up
Duration	*t* / h	1
End conditions	-	Case 2.1
H_2_O conditions		

Subcritical pressure	$p_{H2O}^{in}$ / bar	140.0
Temperature	$T_{H2O}^{in}$ / °C	25.0
Mass flow rate	$T_{H2O}^{out}$ / kg/s	0.000

Sand conditions		

Temperature	*T*_*sand*_ / °C	25.0
Mass flow rate	${\overset{˙}{m}}_{sand}$ / kg/s	0.000

Geometry		

Heat exchanger design	-	Basic
Vertical rows	*n* / -	14
Horizontal rows	*m* / -	42
Modifications	-	No

Results		

Ledinegg Instabilities	-	Yes
Flow Reversals	-	Yes

**Table 13:** Case 2.4.

Mode	-	Heating
Load	-	Shut-down
Duration	*t* / h	1
Start conditions	-	Case 2.1
H_2_O conditions		

Subcritical pressure	$p_{H2O}^{in}$ / bar	140.0
Inlet temperature	$T_{H2O}^{in}$ / °C	270.3
Outlet temperature	$T_{H2O}^{out}$ / °C	509.5
Mass flow rate	${\overset{˙}{m}}_{H2O}$ / kg/s	0

Sand conditions		

Inlet temperature	$T_{sand}^{in}$ / °C	513.4
Outlet temperature	$T_{sand}^{out}$ / °C	328.7
Mass flow rate	${\overset{˙}{m}}_{sand}$ / kg/s	0

Geometry		

Heat exchanger design	-	Basic
Vertical rows	*n* / -	14
Horizontal rows	*m* / -	42
Modifications	-	No

Results		

Ledinegg Instabilities	-	Yes
Flow Reversals	-	Yes

**Table 14:** Case 3.1.

Mode		Cooling
Load		Full-load
H_2_O conditions		

Supercritical pressure at inlet	$p_{H2O}^{in}$ / bar	240.1
Inlet temperature	$T_{H2O}^{in}$ / °C	434.8
Outlet temperature	$T_{H2O}^{out}$ / °C	349.7
Mass flow rate	${\overset{˙}{m}}_{H2O}$ / kg/s	1.25
Pressure difference heat exchanger	Δ*p*_*H*2*O*_ / Pa	18421.2

Sand conditions		

Inlet temperature	$T_{sand}^{in}$ / °C	99.35
Outlet temperature	$T_{sand}^{out}$ / v	421.8
Mass flow rate	${\overset{˙}{m}}_{sand}$ / kg/s	4.84

Geometry		

Heat exchanger design	-	Basic
Vertical rows	*n* / -	14
Horizontal rows	*m* / -	42
Modifications	-	No

Results		

Transferred heat	$\overset{˙}{Q}$ / kW	15832.5
Unbalance factor	*F*1 / -	0.9945
Absolute nonuniform coefficient	*S* / -	0.00017
Relative nonuniform coefficient	*i* / -	0.0019

**Table 15:** Case 4.1.

Mode		Cooling
Load		Full-load
H_2_O conditions		

Subcritical pressure at inlet	$p_{H2O}^{in}$ / bar	140.8
Inlet temperature	$T_{H2O}^{in}$ / °C	535.3
Outlet temperature	$T_{H2O}^{out}$ / °C	277.1
Mass flow rate	${\overset{˙}{m}}_{H2O}$ / kg/s	0.61
Pressure difference heat exchanger	Δ*p*_*H*2*O*_ / Pa	82376.5

Sand conditions		

Inlet temperature	$T_{sand}^{in}$ / °C	195.0
Outlet temperature	$T_{sand}^{out}$ / °C	379.6
Mass flow rate	${\overset{˙}{m}}_{sand}$ / kg/s	7.0

Geometry		

Heat exchanger design	-	Basic
Vertical rows	*n* / -	14
Horizontal rows	*m* / -	42
Modifications	-	No

Results		

Transferred heat	$\overset{˙}{Q}$ / kW	15832.5
Unbalance factor	*F*1 / -	0.9785
Absolute nonuniform coefficient	*S* / -	0.00031
Relative nonuniform coefficient	*i* / -	0.0071

**Table 16:** Case 5.1.

Mode		Heating
Load		Full-load
H_2_O conditions		

Supercritical pressure at inlet	$p_{H2O}^{in}$ / bar	242.1
Inlet temperature	$T_{H2O}^{in}$ / °C	270.5
Outlet temperature	$T_{H2O}^{out}$ / °C	475.5
Mass flow rate	${\overset{˙}{m}}_{H2O}$ / kg/s	0.61
Pressure difference heat exchanger	Δ*p*_*H*2*O*_ / Pa	5759.4

Sand conditions		

Inlet temperature	$T_{sand}^{in}$ / °C	513.4
Outlet temperature	$T_{sand}^{out}$ / °C	338.7
Mass flow rate	${\overset{˙}{m}}_{sand}$ / kg/s	5.77

Geometry		

Heat exchanger design		BasicA
Vertical rows	*n* / -	14
Horizontal rows	*m* / -	42
Modifications	-	Yes

Results		

Transferred heat	$\overset{˙}{Q}$ / kW	1157.7
Unbalance factor	*F*1 / -	0.9546
Absolute nonuniform coefficient	*S* / -	0.00064
Relative nonuniform coefficient	*i* / -	0.0147

**Table 17:** Case 6.1.

Mode		Heating
Load		Full-load
H_2_O conditions		

Supercritical pressure at inlet	$p_{H2O}^{in}$ / bar	241.9
Inlet temperature	$T_{H2O}^{in}$ / °C	270.5
Outlet temperature	$T_{H2O}^{out}$ / °C	474.9
Mass flow rate	${\overset{˙}{m}}_{H2O}$ / kg/s	1.83
Pressure difference heat exchanger	Δ*p*_*H*2*O*_ / Pa	43258.7

Sand conditions		

Inlet temperature	$T_{sand}^{in}$ / °C	513.4
Outlet temperature	$T_{sand}^{out}$ / °C	338.6
Mass flow rate	${\overset{˙}{m}}_{sand}$ / kg/s	17.31

Geometry		

Heat exchanger design	-	Triple
Vertical rows	*n* / -	42
Horizontal rows	*m* / -	42
Modifications	-	No

Results		

Transferred heat	$\overset{˙}{Q}$ / kW	3469.5
Unbalance factor	*F*1 / -	0.5491
Absolute nonuniform coefficient	*S* / -	0.0078
Relative nonuniform coefficient	*i* / -	0.1795

**Table 18:** Case 6.2.

Mode		Heating
Load		Full-load
H_2_O conditions		

Supercritical pressure at inlet	$p_{H2O}^{in}$ / bar	242.2
Inlet temperature	$T_{H2O}^{in}$ / °C	270.5
Outlet temperature	$T_{H2O}^{out}$ / °C	473.9
Mass flow rate	${\overset{˙}{m}}_{H2O}$ / kg/s	1.83
Pressure difference heat exchanger	Δ*p*_*H*2*O*_ / Pa	62101.4

Sand conditions		

Inlet temperature	$T_{sand}^{in}$ / °C	513.4
Outlet temperature	$T_{sand}^{out}$ / °C	338.4
Mass flow rate	${\overset{˙}{m}}_{sand}$ / kg/s	17.31

Geometry		

Heat exchanger design	-	Triple
Vertical rows	*n* / -	42
Horizontal rows	*m* / -	42
Modifications	-	Yes

Results		

Transferred heat	$\overset{˙}{Q}$ / kW	3461.3
Unbalance factor	*F*1 / -	0.8847
Absolute nonuniform coefficient	*S* / -	0.0016
Relative nonuniform coefficient	*i* / -	0.0361

**Figure 21:**
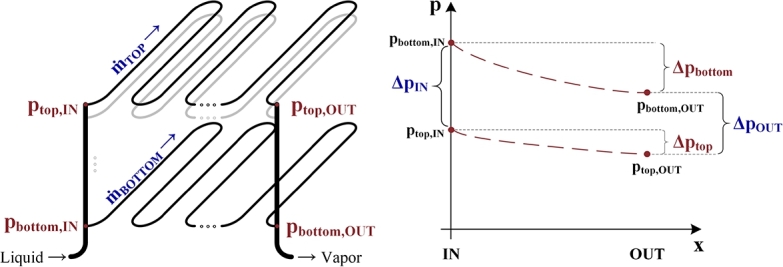
Sketch for understanding pressures and pressure differences for derivation of the unbalance factor *F*1_*approx*_.

## Data Availability

The APROS simulation files are available to download from https://zenodo.org/records/8245847. The results of the simulated cases are available to download from https://zenodo.org/records/8245831.

## Appendix A Abbreviations used in Table 1

See Table 3.

## Appendix B Heat exchanger geometrical data

See Table 4, Table 5.

## Appendix C Simulated cases

See Table 6, Table 7, Table 8, Table 9, Table 10, Table 11, Table 12, Table 13, Table 14, Table 15, Table 16, Table 17, Table 18.

## Appendix D Derivation of unbalance factor $F1_{approx}$

See Fig. 21 and Equations (13)-(24).

Define:

(13) $$\Delta p_{H}=||\Delta p_{IN}|-|\Delta p_{OUT}||$$

(14) $$\Delta p_{H}=p_{bottom,IN}-p_{top,IN}-p_{bottom,OUT}-p_{top,OUT}$$

Rearrange:

(15) $$p_{top,IN}-p_{top,OUT}=-\Delta p_{H}+p_{bottom,IN}-p_{bottom,OUT}$$

Define:

(16) $$\Delta p_{T}=||\Delta p_{bottom,IN}|-|\Delta p_{bottom,OUT}||$$

Define:

(17) $$F1=\frac{{\overset{˙}{m}}_{min}}{{\overset{˙}{m}}_{max}}=\frac{{\overset{˙}{m}}_{top}}{{\overset{˙}{m}}_{bottom}}$$

Bernoulli equation:

(18) $$p_{top,IN}+\rho_{top,IN}⁎\frac{v_{top}^{2}}{2}=p_{top,OUT}+\rho_{top,OUT}⁎\frac{v_{top}^{2}}{2}$$

Use $v=\frac{\overset{˙}{m}}{\rho }$ and rearrange:

(19) $${\overset{˙}{m}}_{top}=\frac{2\Delta p_{top}}{\frac{1}{\rho_{top,OUT}}-\frac{1}{\rho_{top,IN}}}$$

Analog formula for bottom tube:

(20) $${\overset{˙}{m}}_{bottom}=\frac{2\Delta p_{bottom}}{\frac{1}{\rho_{bottom,OUT}}-\frac{1}{\rho_{bottom,IN}}}$$

Use (19) and (20) in (17):

(21) $$F1^{2}=\frac{\Delta p_{top}}{\Delta p_{bottom}}\frac{\frac{\rho_{top,OUT}⁎\rho_{top,IN}}{\rho_{top,IN}-\rho_{top,OUT}}}{\frac{\rho_{bottom,OUT}⁎\rho_{bottom,IN}}{\rho_{bottom,IN}-\rho_{bottom,OUT}}}$$

Assumption (acceptable for superheaters - no two phases):

(22) $$\frac{\frac{\rho_{top,OUT}⁎\rho_{top,IN}}{\rho_{top,IN}-\rho_{top,OUT}}}{\frac{\rho_{bottom,OUT}⁎\rho_{bottom,IN}}{\rho_{bottom,IN}-\rho_{bottom,OUT}}}=1$$

Leads to:

(23) $$F1_{approx}^{2}=\frac{\Delta p_{top}}{\Delta p_{bottom}}$$

Use (15) and (16) in (23) (extracting the root will give Formula (5)):

(24) $$F1_{approx}^{2}=1-\frac{\Delta p_{H}}{\Delta p_{T}}$$
